# Evidence for a novel coding sequence overlapping the 5'-terminal ~90 codons of the Gill-associated and Yellow head okavirus envelope glycoprotein gene

**DOI:** 10.1186/1743-422X-6-222

**Published:** 2009-12-17

**Authors:** Andrew E Firth, John F Atkins

**Affiliations:** 1 BioSciences Institute, University College Cork, Cork, Ireland; 2 Department of Human Genetics, University of Utah, Salt Lake City, UT 84112-5330, USA

## Abstract

The genus *Okavirus *(order *Nidovirales*) includes a number of viruses that infect crustaceans, causing major losses in the shrimp industry. These viruses have a linear positive-sense ssRNA genome of ~26-27 kb, encoding a large replicase polyprotein that is expressed from the genomic RNA, and several additional proteins that are expressed from a nested set of 3'-coterminal subgenomic RNAs. In this brief report, we describe the bioinformatic discovery of a new, apparently coding, ORF that overlaps the 5' end of the envelope glycoprotein encoding sequence, ORF3, in the +2 reading frame. The new ORF has a strong coding signature and, in fact, is more conserved at the amino acid level than the overlapping region of ORF3. We propose that translation of the new ORF initiates at a conserved AUG codon separated by just 2 nt from the ORF3 AUG initiation codon, resulting in a novel 86 amino acid protein.

## Findings

**Figure 1:**
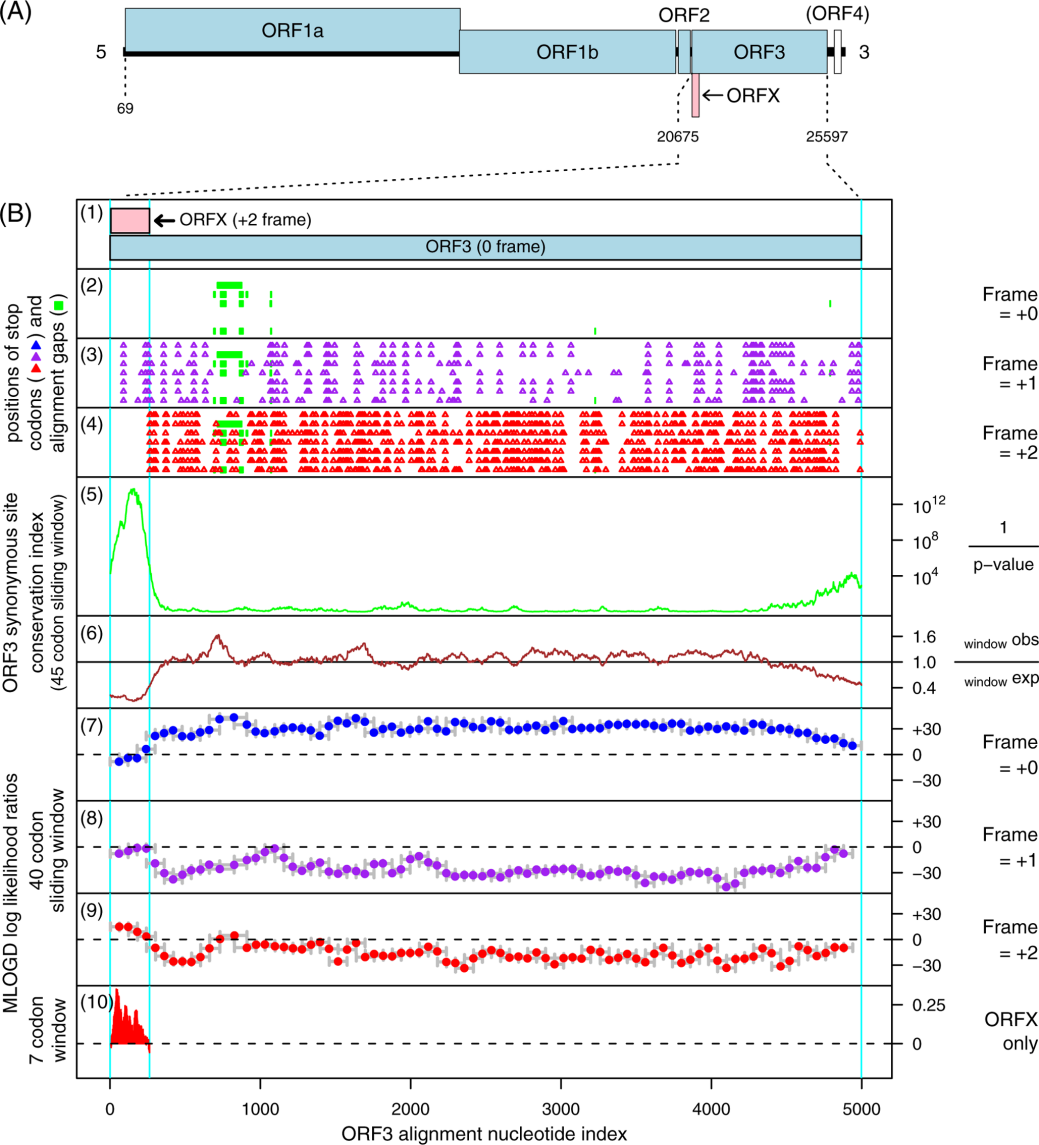
**Coding potential statistics for okavirus ORF3 and the overlapping ORFX**. **(A) **Okavirus genome map (GAV [GenBank:AF227196]). **(B2-B10) **Coding potential statistics based on an alignment of seven okavirus full-length ORF3 sequences (see text for accession numbers). **(B2-B4) **Positions of stop codons in each of the three forward reading frames. Note the conserved absence of stop codons in the +2 frame within ORFX. **(B5-B6) **Conservation at synonymous sites within ORF3 (see [15]). (B5) depicts the probability that the degree of conservation within a given window could be obtained under a null model of neutral evolution at synonymous sites, while (B6) depicts the ratio of the observed number of substitutions within a given window to the number expected under the null model. **(B7-B9) **MLOGD sliding-window plots (see [12]). The null model, in each window, is that the sequence is non-coding, while the alternative model is that the sequence is coding in the given reading frame. Positive scores favour the alternative model and, as expected, there is a strong coding signature in the +0 frame (B7) throughout ORF3 *except *where ORF3 is overlapped by ORFX. In the +1 and +2 frames (B8-B9), scores are generally negative. However, the ORFX region has consecutive high positively scoring windows (B9). **(B10) **MLOGD statistics restricted to ORFX. Here, for increased sensitivity, the null and alternative models were fitted specifically for the ORFX region. The null model is that only the ORF3 frame is coding, while the alternative model is that both the ORF3 frame and ORFX are coding.

The genus *Okavirus *belongs to the family *Roniviridae *in the order *Nidovirales*. Members include Gill-associated virus (GAV) and Yellow head virus (YHV), both of which infect *Penaeus monodon *shrimp. GAV and YHV are currently classified as two of six or more genotypes of the single species *Gill-associated virus *or the Yellow head complex. As with other members of the order *Nidovirales*, these viruses have a linear positive-sense ssRNA genome encoding a large replicase polyprotein that is expressed from the genomic RNA (ORF1a and, via ribosomal frameshifting, an ORF1a-ORF1b fusion product), and a number of other proteins - including the structural proteins - which are translated from a nested set of 3'-coterminal sub-genomic RNAs [1-4]. In the case of the okaviruses, the 3' ORFs encode a polyprotein (ORF3; ~1650 codons) that is cleaved to produce three mature proteins (envelope glycoproteins gp116 and gp64, and an ~25 kDa N-terminal fragment), a nucleocapsid (ORF2; ~145 codons), and possibly an additional short ORF (ORF4) encoded by the sequence 3' of ORF3 (Figure 1A) [4-10].

Overlapping genes are common in RNA viruses where they serve as a mechanism to optimize the coding potential of compact genomes. However, annotation of overlapping genes can be difficult using conventional gene-finding software [11]. Recently we have been using a number of complementary approaches to systematically identify new overlapping genes in virus genomes [11-15]. When we applied these methods to the okaviruses, we found strong evidence for a new coding sequence - hereafter ORFX - overlapping the 5'-terminal 88 codons of ORF3 in the +2 reading frame (Figure 1). Here we describe the bioinformatic analyses.

Okavirus sequences in GenBank with full-length coverage of ORF3 were identified by applying tblastn [16] to the ORF3 amino acid sequence from one GAV isolate [GenBank:AF227196] and one YHV isolate [GenBank:EU487200], resulting in the additional sequences [GenBank:EF156405], [GenBank:FJ848673], [GenBank:FJ194949], [GenBank:EU785042] and [GenBank:EU785043]. The ORF3 regions were extracted, translated, aligned with CLUSTALW [17], and back-translated to give a nucleotide sequence alignment for ORF3.

The alignment was analysed for conservation at synonymous sites, as described in [15]. The procedure takes into account whether synonymous site codons are 1-, 2-, 3-, 4- or 6-fold degenerate and the differing probabilities of transitions and transversions. The analysis revealed a striking, and highly statistically significant (*p *< 10^-21 ^for the total conservation within ORFX), peak in synonymous site conservation at the 5' end of ORF3 (Figure 1B, panels 5-6). Such conservation peaks are indicative of overlapping functional elements, though such elements may be either coding or non-coding. However, in this case, coinciding with the conserved region there was a conserved absence of stop codons in the +2 reading frame (Figure 1B; panel 4), thus suggesting an overlapping coding sequence in the +2 frame as a possible explanation for the enhanced conservation at ORF3-frame synonymous sites.

Inspection of an additional 33 sequences (FJ438530-FJ438532, FJ428584-FJ428613; [18]) with only partial coverage of ORF3, but nearly complete coverage of the ORFX region, again revealed the complete absence of +2 frame stop codons in ORFX. One further partial sequence, [GenBank:DQ978360], differed in having a single nucleotide deletion ~15 codons into ORFX. The effect of this deletion is to fuse the 5' end of ORFX with the downstream ORF3: ribosomes initiating at the ORF3 initiation codon terminate early while ribosomes initiating at the ORFX initiation codon (see below) go on to translate an ORFX-ORF3 fusion. In fact, except for this deletion, the 717 nt DQ978360 is identical to the corresponding region of FJ848673, so it is possible that this deletion is simply due to a sequencing error or a defective sequence.

Given the short length of ORFX, conservation alone is perhaps not sufficient evidence for a coding assignment. Therefore, the alignment was also analysed with MLOGD - a gene-finding program which was designed specifically for identifying overlapping coding sequences, and which includes explicit models for sequence evolution in multiply-coding regions [11,12]. In contrast to the synonymous site conservation index above, MLOGD, when applied in the 'sliding window' mode, does not depend on the degree of conservation *per se *(the sequence divergence parameter is fitted independently for each window). When applied to the ORF3 alignment, MLOGD detected a strong coding signature for ORFX - with positively scoring windows throughout the ORFX region in the +2 frame - indicating directly that ORFX is indeed a coding sequence (Figure 1B, panel 9). In fact, the MLOGD score in the +2/ORFX frame within the ORFX region was significantly greater than the score in the +0/ORF3 frame, indicating that the ORFX product is subject to stronger functional constraints than the product of the overlapping region of ORF3 (which indeed has a negative MLOGD score towards the 5'-terminal half of the ORFX region; Figure 1B, panel 7). Consistently, a comparison of GAV AF227196 with YHV EU487200 showed that, in the region where ORFX and ORF3 overlap, ORFX has higher amino acid conservation than ORF3 (71/86 identities for ORFX, 62/86 identities for ORF3).

When MLOGD was applied in the 'test query ORF' mode (Figure 1B, panel 10), the number of independent base variations across the alignment within the ORFX region was calculated to be N_var _~ 57, and the total MLOGD score was log(LR) ~ 33.0 (see [12] for details). Although MLOGD has a significant false negative rate (i.e. there are known overlapping genes that it fails to detect - particularly ones that are less conserved than the genes they overlap), the false positive rate (with appropriate thresholds) is low. In particular, extensive tests with known single-coding and double-coding virus sequence alignments indicate that 'N_var _≥ 20' and 'log(LR) ≥  × N_var_' signals robust detection (<1% false positive rate) of an overlapping same-strand CDS [12] (and unpublished data).

**Figure 2:**
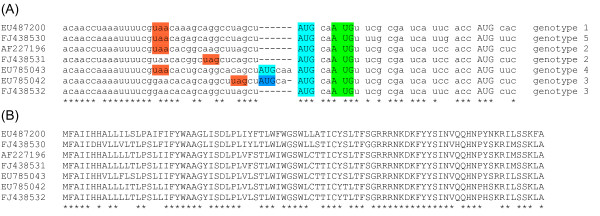
**Nucleotide and amino acid sequence alignments**. **(A) **Nucleotide alignment of the 5'-terminal region of the ORF3 sgRNA. All currently available and non-identical okavirus sequences with coverage of this region are shown. A further four sequences (DQ978360, FJ194949, FJ848673 and EF156405) are locally identical to EU487200. Genotype designations within the Yellow head complex are based on the ORF3 phylogeny of Ref. [18]. Spaces separate +0/ORF3-frame codons. All AUG codons are indicated in capitals. Colour coding is as follows: light blue - ORF3 initiation codon(s); green - proposed +2/ORFX-frame initiation codon; dark blue - upstream +1 frame AUG codon (31 codon ORF); orange - last upstream ORFX-frame stop codons. **(B) **Amino acid alignment of the translated ORFX for representative okavirus sequences.

As of 27 Sep 2009, we located 11 okavirus sequences with coverage of the ORF3 initiation codon, 10 of which also included coverage of the ~35 nt ORF3 sgRNA 5'UTR [8]. Inspection of these sequences showed that all 11 possessed a +2/ORFX-frame AUG codon downstream of, and separated by just 2 nt from, the ORF3 AUG initiation codon (Figure 2A). Both the ORF3-frame and ORFX-frame AUG codons generally have a 'G' at -3 and a pyrimidine at +4, resulting in a medium rather than strong Kozak context. In Turnip yellow mosaic tymovirus, the AUG initiation codons of the overlapping p69 and p206 coding sequences are also closely spaced (4 nt separation). In this case, it has been demonstrated with mutant sequences that, provided the two AUG codons are separated by not more than ~10 nt, the efficiency of initiation at the second AUG codon is greatly enhanced compared with the efficiency obtained for canonical leaky scanning (i.e. for greater separations) given the same Kozak contexts [19]. Moreover, initiation is competitively coupled in the sense that mutation of the downstream AUG codon increases the efficiency of initiation at the upstream AUG codon, and *vice versa*. Thus, initiation at the okavirus ORFX-frame AUG codon is likely to be significantly more efficient than would be expected for normal leaky scanning. Only two of the 11 okavirus sequences have additional upstream AUG codons (Figure 2A). EU785043 has an additional ORF3-frame AUG codon two codons upstream - apparently due to a duplication of the nucleotides 'AUGCAA'. This does not affect our interpretation, however, since the proximity of the ORFX-frame AUG codon to the first ORF3-frame AUG codon (8 nt separation) is within the bounds for the translation initiation coupling mechanism of Ref. [19]. EU785042, on the other hand, has an apparent duplication of the nucleotides 'AUGCA', resulting in an upstream AUG codon in-frame with neither ORF3 nor ORFX. Again all three AUG codons are separated by no more than 7 nt, thus presumably allowing efficient initiation on all three; however, the total number of ribosomes translating ORF3 and ORFX will be depleted due to those that initiate on the first AUG codon (31 codon ORF).

Initiation at the proposed AUG codon would give ORFX the nucleotide coordinates 20680..20937 in AF227196 (GAV) and 20990..21247 in EU487200 (YHV), resulting in an 86 amino acid product with a molecular mass of 10 kDa. The full predicted amino acid sequences are shown in Figure 2B. Application of blastp [16] to the amino acid sequences revealed no similar sequences in GenBank - as expected for a gene created *de novo *via out-of-frame 'overprinting' of a preexisting gene [20,21]. Application of InterProScan [22] predicted an N-terminal signal peptide/transmembrane region, and an additional transmembrane region comprising amino acids 34 to 54. Application of SignalP 3.0 [23] predicted an N-terminal signal anchor or signal peptide. If the latter, cleavage of the signal peptide - mostly probably between residues 25 and 26 - would leave a 61 amino acid, 7.3 kDa ORFX product.

Overlapping genes are difficult to identify and are often overlooked. However, it is important to be aware of such genes as early as possible in order to avoid confusion (otherwise functions of the overlapping gene may be wrongly ascribed to the gene they overlap), and also so that the functions of the overlapping gene may be investigated in their own right. We hope that presentation of this bioinformatic analysis will help fullfil these goals. Initial verification of ORFX product could be by means of immunoblotting with ORFX-specific antibodies.

## Competing interests

The authors declare that they have no competing interests.

## Authors' contributions

AEF carried out the bioinformatic analysis and wrote the manuscript. Both authors edited and approved the final manuscript.
